# Identifying subgroups in heart failure patients with multimorbidity by clustering and network analysis

**DOI:** 10.1186/s12911-024-02497-0

**Published:** 2024-04-15

**Authors:** Catarina Martins, Bernardo Neves, Andreia Sofia Teixeira, Miguel Froes, Pedro Sarmento, Jaime Machado, Carlos A. Magalhães, Nuno A. Silva, Mário J. Silva, Francisca Leite

**Affiliations:** 1 Instituto Superior Técnico, Universidade de Lisboa, Lisboa, Portugal; 2 INESC-ID, Lisboa, Portugal; 3 Hospital da Luz Lisboa, Internal Medicine, Luz Saúde, Lisboa, Portugal; 4 Hospital da Luz Learning Health, Luz Saúde, Lisboa, Portugal; 5 LASIGE and Departamento de Informática, Faculdade de Ciências, Universidade de Lisboa, Lisboa, Portugal

**Keywords:** Cluster analysis, Disease subtyping, Heart failure, Multimorbidity, Phenotyping

## Abstract

This study presents a workflow for identifying and characterizing patients with Heart Failure (HF) and multimorbidity utilizing data from Electronic Health Records. Multimorbidity, the co-occurrence of two or more chronic conditions, poses a significant challenge on healthcare systems. Nonetheless, understanding of patients with multimorbidity, including the most common disease interactions, risk factors, and treatment responses, remains limited, particularly for complex and heterogeneous conditions like HF. We conducted a clustering analysis of 3745 HF patients using demographics, comorbidities, laboratory values, and drug prescriptions. Our analysis revealed four distinct clusters with significant differences in multimorbidity profiles showing differential prognostic implications regarding unplanned hospital admissions. These findings underscore the considerable disease heterogeneity within HF patients and emphasize the potential for improved characterization of patient subgroups for clinical risk stratification through the use of EHR data.

## Introduction

As life expectancy increases, the population suffering from more than one chronic condition is increasing dramatically. The co-occurrence of two or more chronic conditions, defined as multimorbidity, is estimated to affect around 50 million people in the European Union, making it one of the most challenging problems faced by the health sector at the current time [1]. Multimorbidity is a significant healthcare problem, associated with poor health outcomes, poorer quality of life, rapid disease progression, and increased healthcare costs [1, 2].

Heart Failure (HF) is estimated to affect 64.3 million people worldwide. In developed countries, its prevalence is generally estimated at 1% to 2% of the general adult population [3]. Despite significant improvements over the last years, HF prognosis remains poor and patients’ quality of life remains low [4]. The current approach to managing HF relies on phenotyping patients according to Ejection Fraction (EF), a measure of cardiac function that categorizes patients into distinct groups with prognostic and therapeutical implications [5]. This is, however, an oversimplification of a complex phenomenon. In recent years, several attempts were made to better characterize HF population regarding etiology, symptoms and comorbidities [6, 7].

The Electronic Health Record (EHR) is the tool for capturing patients’ medical history, containing structured data, such as laboratory results and diagnoses, along with unstructured data, such as radiology reports, discharge summaries, and other clinical narratives. EHRs, therefore, present a rich data source of patients with complex and heterogeneous conditions, such as HF, COPD (Chronic Obstructive Pulmonary Disease), Dementia, and Parkinson’s Disease, leading to important insights regarding disease pathophysiology [7]. The characterization of patient cohorts through their phenotypes not only better elucidates individual conditions but can also provide a better understanding of the most common associations and interactions between diseases, as well as improving clinical risk assessment [8, 9]. Disease sub-typing is also important for drug development and clinical trial recruitment strategies [10].

There are several approaches to phenotyping the EHR. In recent years, research has been shifting to machine learning methodologies, including unsupervised learning methods that require no prior classification and are truly *data-driven* [10]. One of the challenges of applying cluster analysis to EHR data is the mixture of categorical and continuous data [11]. Among previous methods to tackle mixed-type data is the use of hybrid distance approaches, that is, using specific distance functions prepared for mixed-type data before applying clustering, such as Gower’s Distance [12]. Other approaches include performing data transformations, such as discretization or dimensionality reduction, using Factor Analysis of Mixed Data (FAMD) [13].

Cluster interpretation in clinical context is a complex task. For instance, clustering data with a high number of features turns interpretation and visualization difficult. Graph visualizations are a simple representation of how entities connect and interact with each other and several previous works explore clustering over graph representations [14–16]. When applied to clinical data, graphs can provide a better understanding of patient characteristics. Phenotypic Disease Networks (PDNs) are a network representation of comorbidities that can be used to study their associations, differences in phenotypes between patients, and disease progression. In a PDN, nodes represent diseases and weighted edges represent links between diseases. The weight can be quantified using different measures, such as co-occurrence frequency or Pearson correlation. These networks have the ability to reveal non-obvious relationships between comorbidities that could bring up important information to improve patient treatment approaches [17].

Another main challenge of finding patient clusters is assessing their clinical implications, including prognostic information. Survival analysis is used for comparing the risk for an event of interest for different groups [18]. Commonly used statistical tests include the estimation of the survival curve using the Kaplan-Meier model, the statistical comparison of two groups using the log-rank test, and the possibility of incorporating additional variables through Cox’s hazards model [19].

This study presents a comprehensive workflow for the identification and characterization of HF patients by harnessing the power of EHR data and employing advanced machine learning methodologies. Our approach seeks to enhance the clinical interpretation of derived findings, enabling healthcare professionals to better understand disease associations, interactions, and progression. By leveraging the rich information contained within EHRs, we aim to uncover novel insights into disease pathophysiology and patient phenotypes, ultimately contributing to improved risk assessment, treatment strategies, and overall patient outcomes. The proposed workflow not only holds promise for advancing our understanding of HF but also serves as a valuable framework for investigating other complex and heterogeneous conditions, paving the way for more personalized and effective healthcare in the face of the growing challenge of multimorbidity.

## Methods

**Fig. 1:**
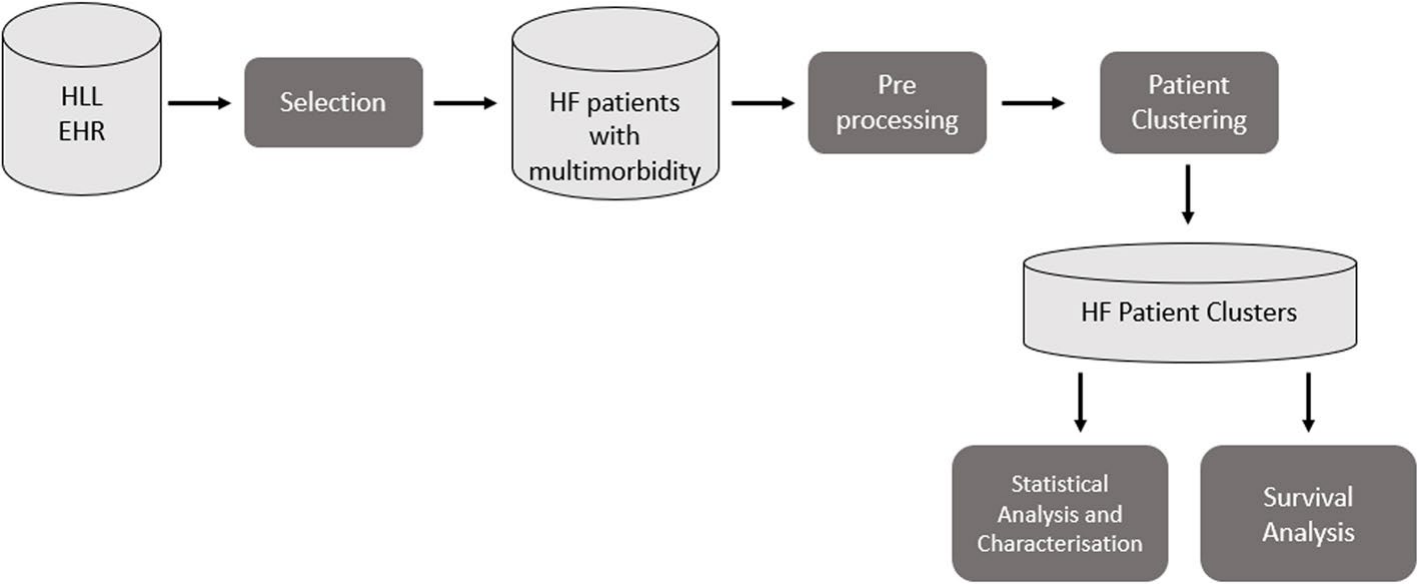
Overview of the proposed approach for the identification and characterization of HF patient subgroups using data from Hospital da Luz Lisboa (HLL)

We developed a workflow for the identification and characterization of HF patient subgroups. The workflow consists of the following steps (see Fig. 1): (1) preprocessing of the data and exploratory data analysis to characterize the dataset; (2) clustering; (3) statistical analysis for characterization and visualization of the obtained clusters, (4) survival analysis to stratify the patient clusters according to the risk for a given outcome.

### Data

We developed the workflow using real-world data from Hospital da Luz Lisboa, Portugal (HLL), the largest private hospital in Portugal. HLL is a university hospital with availability of all medical specialties. Despite being a tertiary hospital, it also has significant primary care activity with a Family Medicine department, covering the full spectrum of care for patients with multimorbidity. The dataset used to develop the pipeline was generated from the EHR of HLL. We used an initial population of 54 827 patients, with an observation period between January 2007 and August 2021. From this initial pool, 3 745 HF patients with multimorbidity were identified using a locally developed phenotyping algorithm that used both ICD-9-CM does and free text mentions from the field “diagnostics and symptoms” from our EHR system (see Table 1). For this algorithm we used keywords that directly refer to HF name, abbreviation or New York Heart Association (NYHA) mention, which is a commonly used staging scale to classify HF symptoms

**Table 1:** ICD-9 Codes and keywords used to identify HF patients. ICFEp - Insuficiência Cardíaca com Fração de Ejeção Preservada, ICFEr - Insuficiência Cardíaca com Fração de Ejeção Reduzida, IC - Insuficiência Cardíaca, ICC - Insuficiência Cardíaca Congestiva, NYHA - New York Heart Association

ICD-9 Codes	428, 398.9.1, 402.0.1, 402.9.1, 404.0.1, 404.0.3, 404.1.1, 404.1.3, 404.9.1, 404.9.3, 425.4, 425.5, 425.6, 425.7, 425.8, 425.9
Keywords	Insuficiência cardíaca, Insuficiência cardiaca, Insuficiencia cardiaca, Insuficiencia cardíaca, ICFEp, ICFEr, IC, ICC, NYHA

**Table 2:** Feature summary of the HF dataset

Phenotypic Domain	Phenotypes	Notes
Demographics	**Age**, **Gender**	Value refers to the last year of observation
Physical Characteristics	**Body mass index (BMI)**	Values are the average of all observations
Lifestyle	Drug use, Alcohol use, Smoking habits	Value refers to the last year of observation
Laboratory	**Sodium**, Potassium, Bicarbonate, **Urea**, **Creatinine**, GFR, Fasting Glucose, **Hemoglobin**, **Platelet count**, **RDW**, **NT-proBNP**, Ferritin, Uric Acid, Sedimentation Rate	Values are the average of all observations.
Comorbidities	**Ischemic Cardiomyopathy**, **Hypertension**, **Diabetes**, **Atrial Fibrillation**, **Cerebrovascular Disease**, **Valvular Disease**, **Chronic Kidney Disease**, **Anaemia**, **Chronic Obstructive Pulmonary Disease**, **Obesity**	Value refers to the last year of observation
Patient complexity	**Number of non-chronic diseases**, **Number of chronic diseases**, **Number of ICD-9 codes**, **Number of consultations**	Value refers to the last year of observation

For the clustering analysis, relevant features were selected based on prior literature review and clinical expertise. These features consisted of clinical variables, demographics, physical characteristics, laboratory data prescriptions, and the most common comorbidities associated with HF, amounting to a total of 35 features (see Table 2). Lifestyle-related features and gender were provided as text fields, comorbidities as binary variables based on the presence or absence of the disease, and all others as numeric. In addition to the features used for clustering, we also extracted the date of HF diagnosis and gathered data on other comorbidities, relevant drug prescriptions, and clinical outcomes. Clinical outcomes evaluated in this study were unplanned hospital admissions: hospitalizations and emergency department visits. Prescription data spans a period of 9 years and 8 months, ranging from January 2012 until August 2021. We grouped drug prescriptions into pharmacological groups that are relevant for HF treatment: ACEis/ ARBs, Beta-blockers, Diuretics, Digoxin and MRA [20]. We also analyzed other groups that are relevant for the most common comorbidities: anticoagulants, antiplatelets, statins and bronchodilators. The number of different drugs is relative to the observation period for medical prescriptions (9 years and 8 months). Percentages of drug groups represent the percentage of patients that had at least one prescription of the relevant medication group during the period analyzed.

### Preprocessing

Prior to the clustering, it was necessary to preprocess the dataset obtained from the EHRs extraction. Categorical features were converted into numeric binary features and features with a prevalence lower than 2% in the cohort were removed. Features with a percentage of missing values higher than 40% were deleted. We utilized the age of patients as recorded in their final observation. Regarding BMI and laboratory results we compiled the mean value for data analysis purposes. This decision was made to mitigate the risk of bias that might arise from relying solely on recent values, which could potentially be anomalous or not reflective of the patient’s typical health status. We employed two imputation methods that were previously shown to have the least imputation error and prediction difference when applied to laboratory data: missForest and multivariate imputation by chained equations (MICE) [21]. Missing values were imputed using Python’s function *Iterative Imputer*, which is based on the MICE method. The MICE method models the missing values of each feature as a function of other features [22]. To do that, at each step, one of the feature columns is designated output *y* and the rest of the feature columns are designated as inputs *x*. Comorbidities were identified using ICD-9 codes. Additionally, we used laboratory data and BMI to increase the sensitivity of anemia and obesity phenotyping, respectively. Continuous features were normalised to have a mean of 0 and a standard deviation of 1. Categorical binary features were scaled from $\{0,1\}\rightarrow \{-0.5,0.5\}$. After preprocessing, the total number of features used for clustering was 25. The features are identified in Table 2 in bold.

### Patient clustering

To apply clustering to the HF dataset, it was first necessary to determine which clustering algorithm and possible complementary techniques to use, and how to evaluate the clustering. It also was necessary to take into account that the dataset was composed of both numerical and categorical data. Several clustering algorithms were tested to understand which one would be more suitable for the HF data. Based on the literature regarding clustering mixed-type data and on HF clustering, we tested the following combinations of methods:

1. Gower’s distance matrix [12] together with Ward’s Agglomerative Hierarchical Clustering [23],
2. dimensionality reduction followed by Ward’s Agglomerative Hierarchical Clustering [23];
3. dimensionality reduction followed by K-Means [24].

Gowers distance is a metric that measures the similarity of two items with mixed numeric and non-numeric data [12]. The Gower distance for instances *x* and *y* is given by:

1 $$\begin{aligned} d_G(x, y) = \frac{\sum _{j=1}^{m} w_j \cdot f_j(x_j, y_j)}{\sum _{j=1}^{m} w_j} \end{aligned}$$

with

$$\begin{aligned} f_j(x_j, y_j) = \left\{ \begin{array}{ll} \frac{|x_j - y_j|}{r_j}, &{} \text {if}\ x_j\ \text {and}\ y_j\ \text {are interval scales} \\ 1 \text { if } x_j \ne y_j, &{} \text {if}\ x_j\ \text {and}\ y_j\ \text {are categorical scales} \end{array}\right. \end{aligned}$$

where *m* is the number of features, *wj* is the feature weight and *I* is the indicator function, that is, *I* is 1 if *xj* and *yj* are equal and 0 otherwise. The weights *wj* were considered 1 for all features.

The dimensionality reduction method chosen was Factor Analysis of Mixed Data (FAMD), a principal component method specific to analyse quantitative and qualitative variables [13]. The FAMD algorithm can be seen as a mix between Princiapl Componente Analysis (PCA) and Mulitple Correspondence Anaoysis (MCA), as it acts as PCA quantitative variables and as MCA for qualitative variables [25].

Given that we were conducting an exploratory analysis without ground truth labels, we could rely solely on the data being clustered and compute internal validity indices for the resulting labels. The used indices were Silhouette Score, Calinski-Harabasz and Davies-Bouldin [26–28]. After computing the three indices for the different clustering algorithms and different values of *k*, we chose the best method using a majority vote (i.e. the algorithm and *k* that performed best in at least two of the indices). Additionally, a minimum of $N > 375$ was also defined to promote stability and ensure that none of the clusters had less than 10% of the total population, an approach that was previously employed by others [7].

### Statistical analysis and characterization of obtained clusters

Clusters were characterized according to age, gender, and comorbidities. Demographic, clinical, and laboratory characteristics were compared using Chi-squared tests for categorical variables and Kruskal-Wallis test for continuous variables. Differences were considered statistically significant whenever *p*-value was inferior to 0.05.

To enhance the interpretation and clinical meaning of the derived findings, we developed a visualization of the clusters’ comorbidities prevalence and associations through graph representation of each cluster. Each graph’s node represents a disease in the cluster and an edge represents a co-occurrence of the two nodes (diseases) connected by that edge. The graphs were created with Python’s *NetworkX* package and *Gephi* for visualization. The settings were adjusted so that node size was proportional to the number of connections to other nodes (node degree) and edge thickness was proportional to disease co-occurrence prevalence (edge weight). Co-occurrences (edges) with a co-occurrence prevalence lower than 2% were discarded to declutter the visualisation. Graph representation of comorbidities provides a better understanding of which diseases co-occur more frequently and which diseases have the most connections with other diseases.

### Survival analysis

We conducted a survival analysis to assess outcome differences among the derived clusters. Survival curves and Hazards Ratio (HR) were computed for unplanned hospital admissions (hospitalizations and emergency department visits), for each cluster [18]. To achieve this, patients were temporally aligned at the starting point $t_{0}$, the moment of HF diagnosis. Time intervals between HF diagnosis and outcomes were subsequently derived. Patients who did not experience the outcome were censored. Analysis was performed using Python’s package *Lifelines*, to create Kaplan-Meier curves and Cox proportional regression models. Kaplan-Meier curves were computed for each outcome individually and were stratified per cluster, with differences between groups tested using the log-rank test. For Cox proportional regression we adopted a previously reported strategy by using three different models for each outcome [29]: an unadjusted model that only took into account the clusters, a second model adjusted for age and gender, and a third model adjusted for age, gender and the laboratory value NT-proBNP, which is a risk marker for HF prognosis. HR from Cox regression models are presented in relation to the lowest risk cluster (determined by the lowest percentage of outcomes).

## Results

### Description of heart failure population

**Fig. 2:**
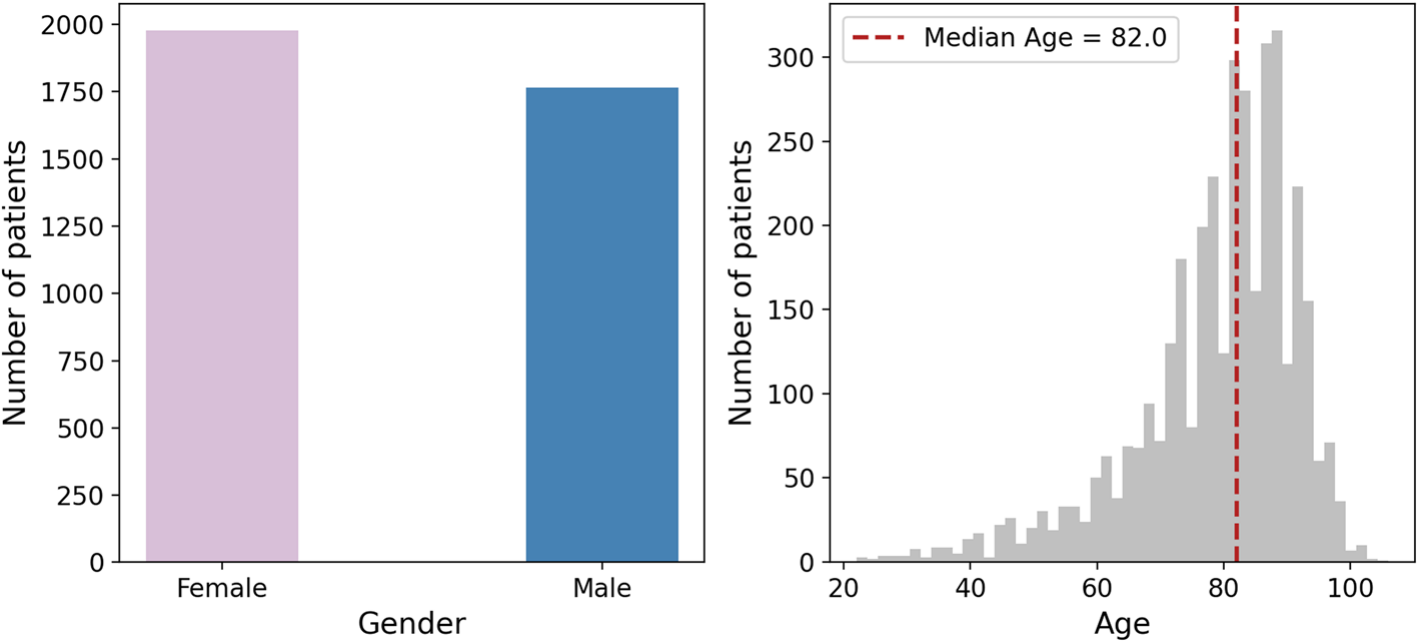
Gender and age distribution of the dataset of HF patients with multimorbidity

The studied population included 3,745 patients who had HF and at least another comorbidity (i.e. multimorbidity patients with HF). The median age was 82 years (IQR 73-88), and 52.84% were women (Fig. 2). The median number of chronic diseases was 5 (IQR 3-7) with approximately 40% of the population having between 3 and 5 comorbidities and approximately 30% having 6 to 8 comorbidities.

**Fig. 3:**
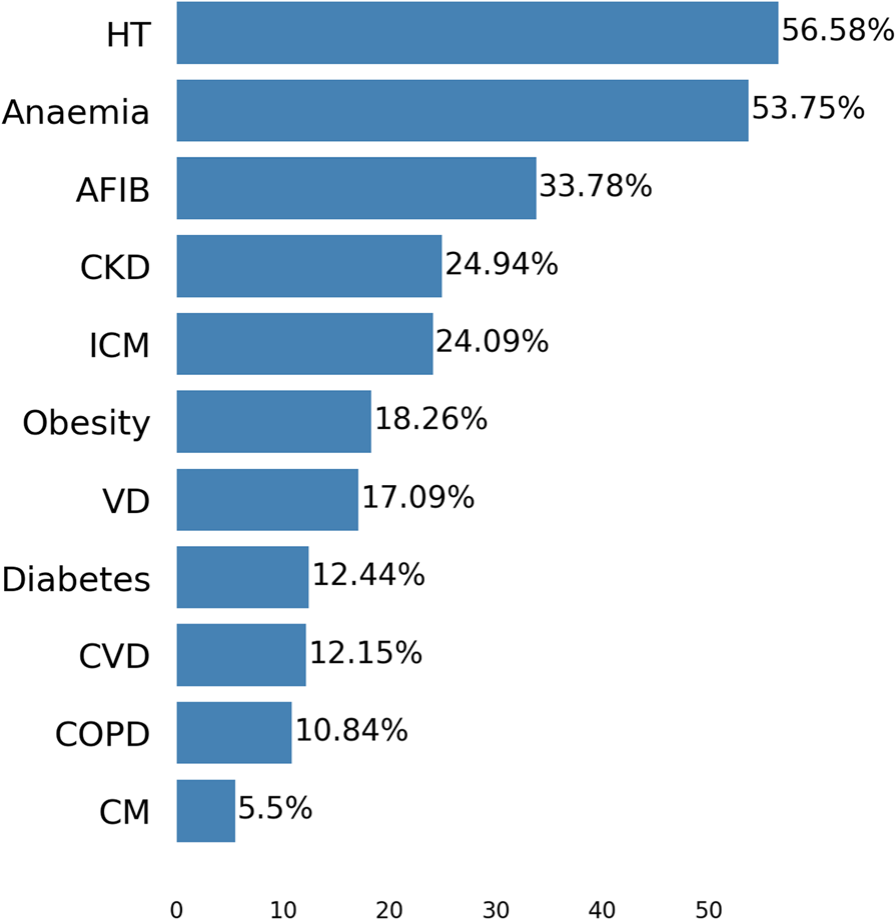
Prevalence of comorbidities used for clustering the HF population. Abbreviations: HT for Hypertension, AFIB for Atrial Fibrillation, CKD for Chronic Kidney Disease, ICM for Ischemic Heart Disease, VD for Valvular Heart Disease, CVD for Cerebrovascular Disease, COPD for Chronic Obstructive Pulmonary Disease, and CM for Other Cardiomyopathies

**Fig. 4:**
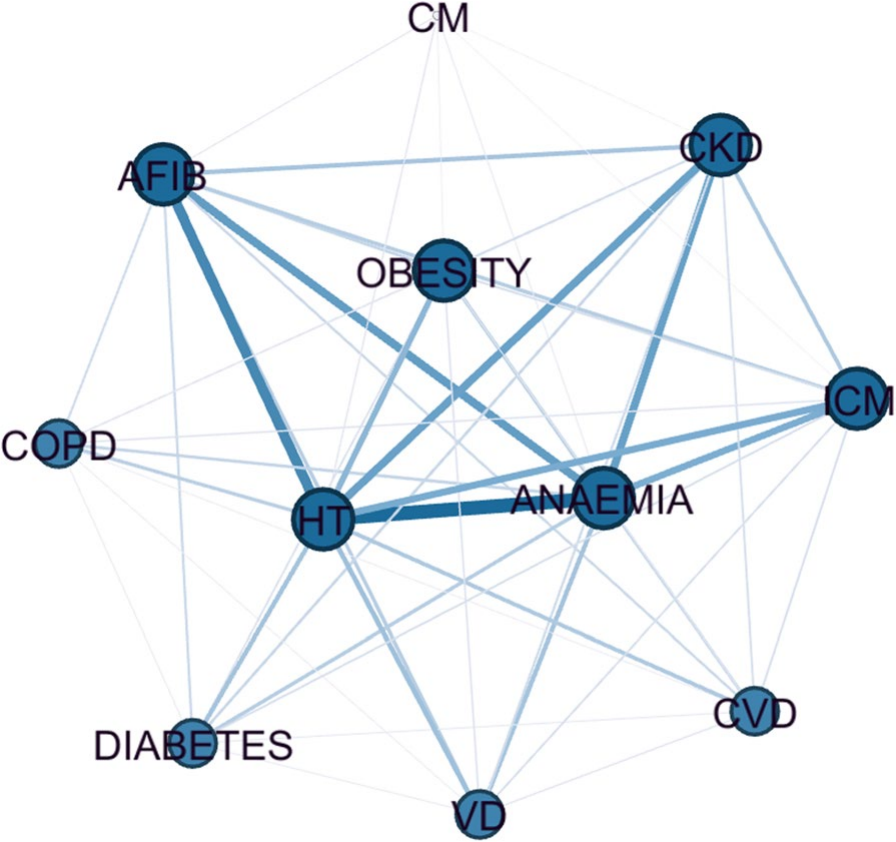
Graph representation of comorbidities used for clustering. In the graph a node represents a disease and its size is proportional to the node degree. An edge represents a co-occurrence of two diseases and its width is proportional to the prevalence of the co-occurrence in the dataset. Using this visualisation, it is possible to obtain extra insights into the relationship between comorbidities, as it is possible to observe which diseases tend to co-occur more frequently. The thickness of the edges makes it possible to verify that HT and Anaemia, and HT and AFIB occur frequently together. CKD and ICM also show a high co-occurrence with HT and Anaemia. ICM–Ischaemic Cardiomyopathy, HT–Hypertension, AFIB–Atrial Fibrillation, CVD–Cerebrovascular Disease, VD-Valvular Disease, CKD–Chronic Kidney Disease, COPD–Chronic Obstructive Pulmonary Disease, CM–Other Cardiomyopathies

The most common comorbidity in the dataset was Hypertension (56.58%), followed by Anaemia (53.75%) and Atrial fibrillation (33.78%). Other highly prevalent conditions were Chronic kidney disease (24.94%) and Coronary artery disease (24.09%) (Fig. 3). In addition to assessing the prevalence of each comorbidity, a graph representation of the comorbidities was also computed (see Fig. 4). In this analysis, Hypertension, Atrial fibrillation and Coronary artery disease are the conditions with the highest prevalence and that co-occur most frequently with other diseases.

### Clustering algorithm and choice of *k*

Taking into account the mixed-type nature of the data (containing both binary an continuous variables), we explored three different clustering approaches and results are summarized in Table 3. We found that the combination of Gower’s distance matrix and Ward’s Hierarchical Agglomerative Clustering was the one that produced clusters with higher scores in all clustering metrics analyzed.

**Table 3:** Values obtained for clustering metrics Silhouette Score. Calinski-Harabasz Index and Davies-Bouldin Score for the different clustering algorithms, namely, Gower’s Distance and Hierarchical Clustering, FAMD and Hierarchical Clustering, and FAMD and K-Means. For Silhouette Score and Calinski-Harabasz a higher value indicates a better performance, for Davies-Bouldin a lower value is best

Clustering Algorithm	k	Silhouette Score	Calinski-Harabasz	Davies-Bouldin
Gower Distance + Hierarchical Clustering	3	0.153	918.620	1.859
	4	0.155	910.162	1.751
	5	0.153	810.238	1.797
FAMD + Hierarchical Clustering	3	0.080	221.906	2.267
	4	0.082	225.429	2.325
	5	0.075	227.625	2.332
FAMD + K-Means	3	0.073	217.615	2.374
	4	0.078	201.213	2.420
	5	0.068	191.439	2.537

After defining the clustering method, the choice of the number of clusters (*k*) was performed by applying Silhouette Score, Calinski-Harabasz, and Davies-Bouldin. We used a minimum of 375 patients to promote stability and performed metrics scores for each value of *k* so that a majority vote (i.e. the best value of *k* with the highest score in the majority of the metrics) is considered. Table 4 shows the clustering evaluation metrics for clusters with $k \in [2,12]$. We found that $k=4$ was the best value across clustering metrics and clinical interpretability. Although $k=2$ had a slighter better score, it produced two asymmetric clusters where one cluster had the majority of the patients and the other a minority (under 375) that also did not differ significantly in most measured attributes. Therefore, the value chosen for the analysis was $k=4$.

**Table 4:** Values obtained for clustering metrics Silhouette Score. Calinski-Harabasz Index and Davies-Bouldin Score for Hierarchical Clustering with Gower’s Distance using k=[4]. For Silhouette Score and Calinski-Harabasz a higher value indicates a better performance, for Davies-Bouldin a lower value is best

Clusters	Silhouette Score	Calinski-Harabasz	Davies-Bouldin
2	0.273	1147.505	1.494
3	0.153	918.620	1.859
**4**	**0.155**	**910.162**	**1.751**
5	0.153	810.238	1.797
6	0.147	746.957	1.739
7	0.141	673.593	1.983
8	0.127	615.439	2.005
9	0.125	571.963	2.041
10	0.130	538.409	1.939
11	0.132	512.288	1.961
12	0.140	491.961	1.914

### Multimorbidity characterization

**Fig. 5:**
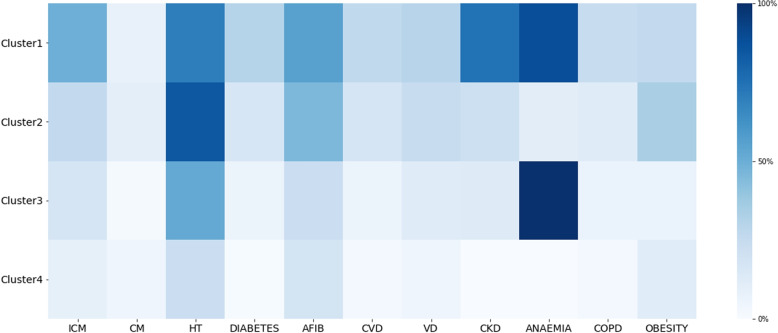
Cluster-specific percentages of comorbidities. A darker color indicates a higher percentage of the comorbidity in the cluster. ICM-Ischaemic Cardiomyopathy, HT-Hypertension, AFIB-Atrial Fibrillation, CVD-Cerebrovascular Disease, VD-Valvular Disease, CKD-Chronic Kidney Disease, COPD-Chronic Obstructive Pulmonary Disease

Figure 5 shows a tileplot of cluster-specific percentages of comorbidities that allows to easily find the most prevalent comorbidities in each cluster while comparing the prevalence of diseases among clusters. Table 5 shows the detailed characterization of each cluster and of the HF dataset. Cluster1 included older male patients with more chronic conditions. Anemia, CKD, and Hypertension were particularly prevalent. In addition, this was the group with the highest values of NT-proBNP. Differently, Cluster2 was characterized by elder women, had highly prevalent Hypertension, AF, and Obesity, and the median NT-proBNP value was the lowest among the four clusters. Cluster3 was the largest one (*n*=1 231), had male predominance (59.46%), and was characterized by an almost universal prevalence of Anemia (99.35%), despite the lower number of prevalent comorbidities compared to the other clusters. Cluster4 had the lowest median age and a female preponderance (73.92%). This cluster was also the cluster with the lowest number of comorbidities.

**Table 5:** Characterisation of the four resulting clusters obtained from HF dataset. Continuous variables are described as median (inter-quartile range) and categorical variables as %. *p*-values for the comparison of the characteristics across clusters

Characteristics	Cluster1	Cluster2	Cluster3	Cluster4	Dataset	*p*-value
Number of Patients	789	866	1231	859	3745	-
Female, %	37.39	63.51	40.54	73.92	52.84	< 0.01
Male, %	62.61	36.49	59.46	26.08	47.16	< 0.01
Age, years	85.0(78.0-89.0)	81.0(73.0-87.0)	83.0(76.0-89.0)	76.0(59.5-85.0)	82.0(73.0-88.0)	< 0.01
BMI, kg/m^2^	24.69(23.31-29.0)	26.14(24.16-31.09)	24.27(23.21-25.7)	24.83(23.44-27.06)	24.8(23.44-27.78)	< 0.01
Ischemic Cardiomyopathy, %	49.43	25.87	17.3	8.73	24.09	< 0.01
Cardiomyopathy, %	7.73	9.93	1.79	4.31	5.5	< 0.01
Hypertension, %	70.22	84.53	52.07	22.35	56.58	< 0.01
Diabetes, %	30.42	16.17	6.17	1.16	12.44	< 0.01
Atrial fibrillation, %	55.64	45.5	22.5	18.04	33.78	< 0.01
Transient Ischemic Attack, %	26.62	17.55	5.85	2.44	12.15	< 0.01
Valvular Disease, %	29.91	24.6	12.19	4.77	17.09	< 0.01
Chronic Kidney Disease, %	74.52	21.48	12.75	0.35	24.94	< 0.01
Anaemia, %	88.47	10.62	99.35	0.0	53.75	< 0.01
COPD, %	24.59	12.47	6.66	2.56	10.84	< 0.01
Obesity, %	25.86	33.95	6.99	11.64	18.26	< 0.01
Number of ICD-9 Codes	14.0(8.0-23.0)	10.0(6.0-16.0)	5.0(2.0-9.0)	4.0(2.0-7.0)	7.0(3.0-13.0)	< 0.01
Number of Chronic Diseases	8.0(6.0-10.0)	6.0(5.0-8.0)	4.0(2.0-5.0)	3.0(2.0-4.0)	5.0(3.0-7.0)	< 0.01
Sodium, mEq/L	140.0(137.0-143.0)	140.0(138.0-142.0)	139.56(137.0-142.0)	139.51(139.0-141.0)	139.57(138.0-142.0)	< 0.01
Urea, mg/dL	81.0(53.0-120.0)	51.0(39.0-68.0)	56.64(43.0-78.0)	44.94(35.78-55.67)	54.0(40.0-79.0)	< 0.01
Creatinin, mg/dL	1.68(1.22-2.36)	1.1(0.89-1.34)	1.23(0.98-1.52)	1.01(0.83-1.16)	1.17(0.93-1.56)	< 0.01
Hemoglobin, g/dL	10.7(9.3-11.9)	13.3(12.1-14.4)	11.0(9.8-12.3)	13.4(12.47-14.2)	12.19(10.6-13.4)	< 0.01
Red Cell Distribution Width, %	15.7(14.4-17.3)	14.39(13.4-15.3)	14.89(14.3-16.3)	14.3(13.4-14.75)	14.69(13.8-15.9)	< 0.01
Platelet count, x10^3^/L	209.0(152.0-264.0)	214.0(172.0-253.81)	225.0(182.0-276.0)	238.0(193.5-264.0)	223.0(176.0-263.08)	< 0.01
NT-proBNP, pg/ml	4873.0(1648.0-13342.0)	1440.5(402.5-4083.19)	3817.0(1455.0-6973.7)	2173.0(531.5-4275.74)	2800.1(942.0-6255.0)	< 0.01
Number of Consultations/year	4.5(1.0-12.5)	3.5(1.0-8.5)	01.0(0.0-3.5)	1.0(0.0-3.5)	2.0(0.5-6.0)	< 0.01

**Fig. 6:**
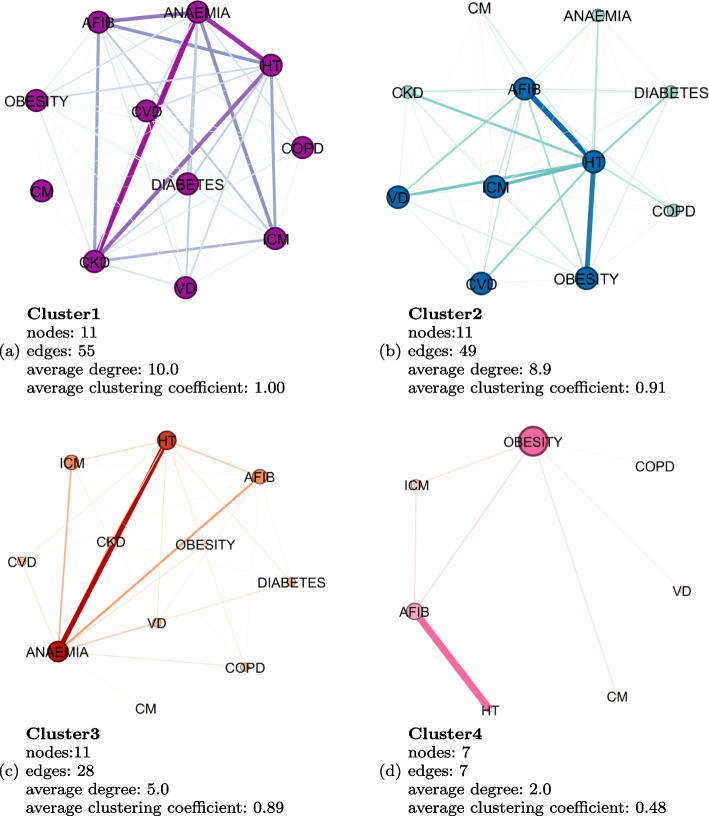
Prevalence and graph representation of comorbidities used for clustering in the HF dataset. In the graph a node represents a disease and its size is proportional to the node degree. An edge represents a co-occurrence of two diseases and its width is proportional to the prevalence of the co-occurrence in the dataset. ICM-Ischaemic Cardiomyopathy, HT-Hypertension, AFIB-Atrial Fibrillation, CVD-Cerebrovascular Disease, VD-Valvular Disease, CKD-Chronic Kidney Disease, COPD-Chronic Obstructive Pulmonary Disease, CM-Other Cardiomyopathies

Network analysis provides additional insight regarding multimorbidity and cluster complexity in HF patients, both through visual inspection of graphs and by comparing average degree (average number of other diseases that are connected to one disease) and average clustering (measure of density that indicates the degree to which nodes in a graph tend to cluster together) coefficients. The clustering coefficient is a measure that quantifies the tendency of nodes in a graph to form clusters or groups of interconnected neighbors. It helps us understand the local structure of the network by assessing how likely it is for neighboring nodes to also be connected to each other [14]. Figure 6 shows the graph representation of each cluster’s comorbidities. We can see that Cluster1 has the highest number of nodes (11) and edges (55), while Cluster4 has the lowest (7), therefore indicating that patients from the first cluster have higher complexity than patients from the former. The most common comorbidity associations are also depicted. For instance, Cluster1 has a high average degree of 10, meaning that all diseases are connected to each other, while also having strong associations between diseases. Cluster2 also has a high average degree of 8.9, whereas Cluster3 and Cluster4 have much lower average degrees (5 and 2, respectively). The fact that all nodes are of similar size means that every disease co-occurs at least once with almost all other diseases. The width of the edges is what allows us to understand which of these co-occurrences, also known as dyads, are more common in each cluster. We can witness that not only comorbidity prevalence differs between clusters, but also dyads exist differently between clusters. For instance, while in Cluster1 the strongest dyads are CKD/Anemia and Hypertension/Anemia, in Cluster2 the most significant dyads are Obesity/AF and Hypertension/AF. Cluster3 has a high number of patients with Hypertension and Anemia, some of them also showing AF, Coronary artery disease, and Valvular heart disease. Cluster4 has a common association between Hypertension and AF, while Obesity is connected to several diseases in the graph but with a lower co-occurrence. The average clustering coefficient is also highest for Cluster1 and lowest for Cluster4 (1 vs. 0.48), which suggests patients from Cluster1 have higher order interactions of diseases than patients from Cluster4.

### Drug prescription analysis

Table 6 shows the mean drug prescriptions of specific pharmacological groups in each cluster. As expected, patients show prescription profiles that are concordant with HF severity and comorbidity profiles. Among all clusters, anticoagulants are the most frequent drug prescription group (47.42%). Cluster1 shows the highest percentage of utilization of all drug groups, including drugs used for treating comorbidities (bronchodilators and hematinic factors), which is both consistent with it being the cluster with most advanced disease stages and the highest number of comorbidities. Patients from Clusters 3 and 4 have a lower percentage of all drug groups when compared to Cluster1 and Cluster2. In Cluster4, despite the high prevalence of Anemia, only 16.37% of patients were prescribed hematinic factors.

**Table 6:** Average presciptions per year and prevalence of prescribed drugs per cluster. *p*-values for the comparison of the characteristics across clusters. ACEi - angiotensin-converting enzyme inhibitor; MRA - Aldosterone receptor antagonists; DPP4i - Dipeptidyl peptidase-4 inhibitor

Medications	Cluster1	Cluster2	Cluster3	Cluster4	*p*-value
Patients with drugs prescribed (%)	87.58	84.41	68.97	66.0	<0.01
Avg prescriptions/year	6.6	4.15	2.53	2.00	<0.01
Anticoagulants (%)	42.98	37.76	26.5	19.58	<0.01
Statins (%)	39.94	33.52	21.91	18.34	<0.01
Beta-Blockers (%)	35.31	35.16	22.38	17.28	<0.01
Antiplatelets (%)	34.88	24.62	21.08	10.76	<0.01
Inhalers Bronchodilator (%)	32.27	23.53	16.49	13.76	<0.01
Diuretics (%)	29.52	26.54	15.19	10.58	<0.01
ACEi \ARBs (%)	40.96	28.32	21.29	17.11	<0.01
Hematinic factors (%)	27.79	15.18	16.37	8.47	<0.01
Anticholinergics (%)	23.59	15.73	11.9	8.64	<0.01
MRA (%)	16.06	14.5	12.25	7.58	<0.01

### Unplanned hospital admissions

Overall, 60.91% of the patients had at least 1 hospitalization and 83.71% had at least one emergency admission during the observation period (see Table 7). Cluster1 had the highest rate of unplanned hospital admissions, both aggregated and number of admissions per year. Cluster2 and Cluster3 had lower percentages of unplanned hospital admissions, while Cluster4 had the lowest rate among the four clusters.

**Table 7:** Characterisations of outcomes related variables per cluster and in the entire dataset. Continuous variables are described as median (inter-quartile range) and categorical variables as %. *p*-values for the comparison of the characteristics across clusters

Characteristics	Cluster1	Cluster2	Cluster3	Cluster4	*p*-value
Number of Hospitalisations/year	0.2(0.1-0.4)	0.1(0.0-0.2)	0.1(0.0-0.2)	0.0(0.0-0.1)	<0.05
Hospitalisations within 1 year of HF diagnosis, %	35.23	23.78	27.86	21.18	<0.01
Hospitalisations within time period analysed, %	86.06	68.13	59.46	32.6	<0.01
Number of Emergency Admissions/year	0.6(0.2-1.2)	0.4(0.1-0.8)	0.2(0.1-0.5)	0.1(0.1-0.4)	<0.05
Emergency admissions within 1 year of HF diagnosis, %	52.47	44.34	39.48	36.20	<0.01
Emergency admissions within time period analysed, %	94.55	88.45	79.45	75.09	<0.01

**Fig. 7:**
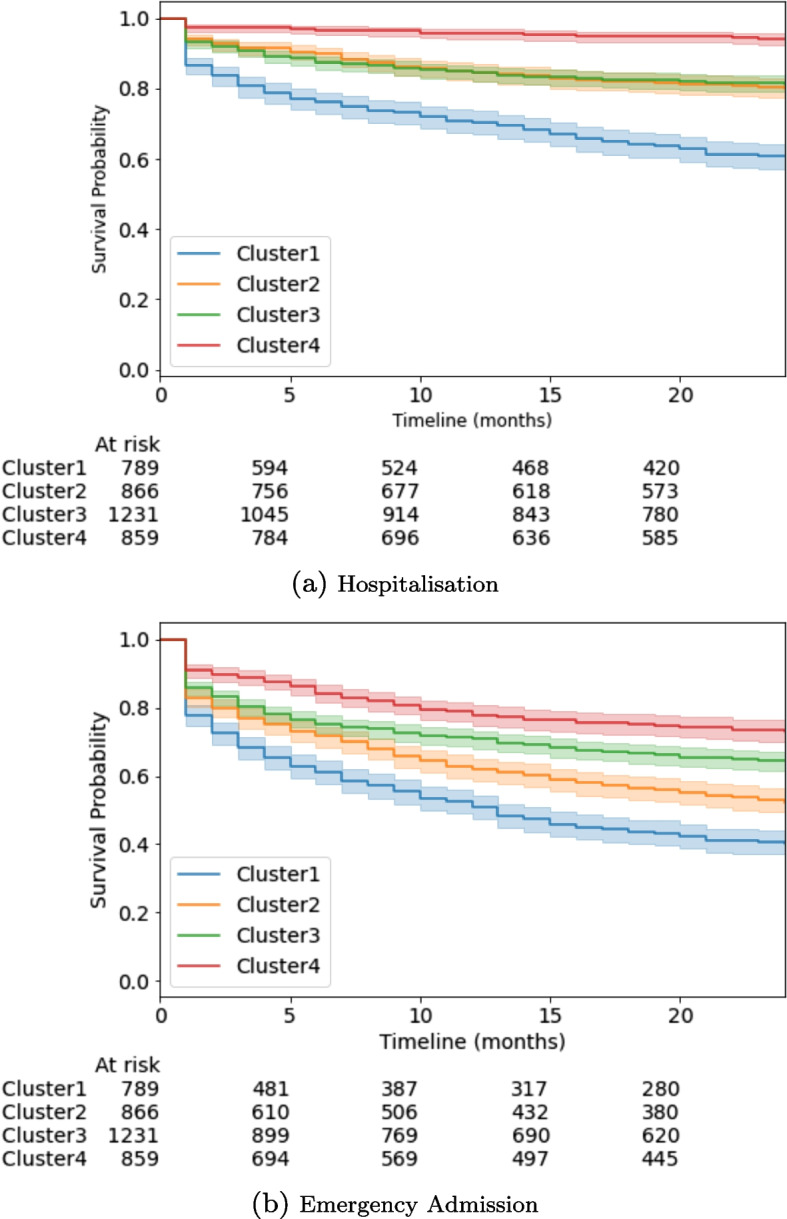
Kaplan-Meier survival curves for the outcomes Hospitalisation and Emergency admission for each cluster (within 2-years after HF diagnosis)

Figure 7 shows univariate Kaplan-Meier curves for the outcomes of hospitalisation and emergency admission, stratified by clusters, for a time period of 2 years post HF diagnosis. Cluster1 appears as the highest risk cluster, having the highest rate of unplanned hospital admissions at all times. Contrarily, Cluster4 shows the lowest rate of unplanned hospital admissions during the study period, while Cluster2 and Cluster3 have in-between values.

Table 8 shows the hazard ratios for three different Cox proportional hazard models computed: Model 1 is an unadjusted model, Model 2 is adjusted for age and gender only and Model 3 is adjusted for age, gender and NT-pro-BNP level. Hazard ratio (HR) is computed in relation to the lowest risk cluster (Cluster4). Cluster 1 shows the highest HR for both emergency department visit (5.86; CI 4.80 - 7.15) and hospital admission (2.73; CI 2.38 - 3.14). Even after adjusting for age, gender, and NT-pro-BNP, differences (Models 2 and 3) in HR among clusters remain significant.

**Table 8:** Risk of clinical events hospitalisation and emergency admission compared with Cluster4 (lowest risk). Hazard ratios and 95% confidence intervals computed using Cox Regression. adjusted for Age, Gender and NT-proBNP (Model3). Model1 adjusted for Age and Gender, Model 2 adjusted for Age, Gender and NT-proBNP

	Cluster1	Cluster2	Cluster3	Cluster4	*p*-value
Model1, HR (95% CI)					
Hospitalisation	5.86 (4.80-7.15)	2.82 (2.29-3.48)	2.43 (1.98-2.98)	1	<0.05
Emergency Admission	2.73 (2.38-3.14)	2.00 (0.84-2.31)	1.29 (1.13-1.49)	1	<0.05
Model2, HR (95% CI)					
Hospitalisation	4.90 (3.97-6.05)	2.57 (2.08-3.18)	2.10 (1.70-2.60)	1	<0.05
Emergency Admission	2.60 (2.24-3.02)	1.92 (1.66-2.21)	1.24 - (1.07-1.44)	1	<0.05
Model3, HR (95% CI)					
Hospitalisation	4.73 (3.83-5.84)	2.58 (2.09-3.19)	2.10 (1.70-2.60)	1	<0.05
Emergency Admission	2.58 (2.22-2.99)	1.92 (1.66-2.21)	1.24 (1.07-1.44)	1	<0.05

## Discussion

The results of this study add to the increasing evidence that HF is a complex syndrome with diverse phenotypes that are partly explained by patterns of multimorbidity. By utilizing clustering and network analysis on a real-world dataset, we identified four distinct clusters of HF patients with differing comorbidity profiles. These clusters exhibited varying disease prevalences and co-occurrence patterns, as well as differences in demographic characteristics and clinical severity, as measured by the risk of unplanned hospital admissions.

We encounter similarities between our findings and previous works. For example, a prior study using model-based clustering on 12 comorbidities of an HF cohort identified five clusters that differed in comorbidities, sociodemographic factors, and prognosis [4]. This study also found a cluster with a worse prognosis and the highest percentage of comorbidities, a cluster with anemia predominance, and lower-burden clusters. Another recent study specifically evaluated multimorbidity profiles in the HF population and identified six different profiles of multimorbidity using exploratory factor analysis, which also had prognostic implications [30]. In our study, Cluster1 had the highest risk profile, with older patients and a greater number of comorbidities. It represents what we call as the Cardio-renal-metabolic syndrome where patients are more likely to be men, have more advanced HF, higher levels of NT-pro-BNP, more severe CKD, and a longer history of Diabetes mellitus. These patients frequently have HF with reduced EF. Cluster2, on the other hand, was slightly younger, had a high prevalence of AF, Obesity, and HT, and showed the second highest risk for the outcome. This pattern clearly resembles one found in the literature with female predominance, obesity, Pulmonary Hypertension, lower levels of NT-pro-BNP and preserved ejection fraction [31]. Cluster3 and Cluster4 showed a better prognosis but differed in the age of patients and the prevalence of conditions.

This study has several strengths, including a novel workflow for phenotyping HF patients through EHR that can be replicated in different settings. We utilized Ward’s Hierarchical Clustering with Gower’s distance, an underexplored clustering algorithm that has advantages on multimodal data such as EHR [12]. Additionally, we employed network analysis metrics, which we believe are highly intuitive for understanding not only the prevalence of comorbidities but also the co-occurrence under different clusters. We utilized a real-world longitudinal database, which allowed us to conduct a survival analysis, presenting advantages over most similar clustering studies that often discretize time and do not account for censoring on outcome measurement [19].

We must acknowledge several limitations in this work. From a methodological perspective, although we chose the optimal number of clusters according to several described metrics, we acknowledge that they are not data-agnostic, and therefore, it may be difficult to compare what constitutes ’good’ clustering across studies [32]. Additionally, as with other studies that make secondary use of EHR data, there are important implicit biases that warrant further validation of findings in different datasets. For example, we make the assumption that the absence of prescribed medications implies that these patients are not currently undergoing any drug treatment. However, it should be noted that we cannot guarantee that they may not have prescriptions for the same drugs at other healthcare facilities, which could potentially impact our conclusions. Although we developed and internally validated a phenotyping algorithm for HF using ICD-9 codes and free text from the field “diagnostics and symptoms”, we understand both false positive and false negative cases might occur, due to wrong data entry under-reporting and and a lack of comprehensiveness in keywords. Using more data modalities and possibly statistical learning methods for phenotyping may improve case detection accuracy. While we measured unplanned hospital admissions as a surrogate for clinical severity and healthcare resource utilization, different outcomes of interest should also be considered, such as mortality and patient-reported outcomes. We also did not include EF information in our data analysis, an essential parameter for establishing the diagnosis and classifying HF. The patients included in this study were identified as having the diagnosis of HF already established but we strongly believe that it would be insightful to analyze EF range of our derived clusters in future work. We intend to develop future work to tackle these limitations.

## Conclusions

This study developed a data workflow to identify and phenotype subgroups of HF patients with multimorbidity, using real-world data from a hospital’s EHR. We identified four clusters that differed in clinical and demographic characteristics, as well as in risk for unplanned hospital admissions. Our findings strengthen the conviction that HF is a complex syndrome with different phenotypes, and that currently available EHR can be utilized to find subgroups with prognostic implications that may be clinically useful for tailoring management. Future work should clarify the relevance of these findings on datasets from other hospitals and through the incorporation of other features extracted from clinical notes and medical imaging (e.g. EF).

## Data Availability

The data used in this study, while obtained from deidentified Electronic Health Records, are subject to strict confidentiality and privacy regulations. Data access requests may be considered on a case-by-case basis and will require approval from the relevant institutional review boards and data custodians. Researchers interested in obtaining access to the data for the purpose of validating or extending the findings presented in this paper should contact the corresponding author for further information on the data access process and the necessary legal and ethical requirements.

## Abbreviations

ACEIs: Angiotensin-converting enzyme inhibitors
ARBs: Angiotensin receptor blockers
BMI: Body mass index
CKD: Chronic kidney disease
COPD: Chronic obstructive pulmonary disease
EHRs: Electronic health records
EF: Ejection fraction
FAMD: Factor analysis of mixed data
HF: Heart failure
HLL: Hospital da Luz Lisboa
HR: Hazards ratio
HT: Hypertension
ICU: Intensive care unit
ICD-9: Internation classification of diseases version 9
IQR: Interquartile range
MICE: Multivariate imputation by chained equations
MRA: Mineralocorticoid receptor antagonists
PDN: Phenotypic disease networks
